# Complete Genome Sequence of a Novel Pseudomonas fluorescens Strain Isolated from the Flower of Kūmarahou (Pomaderris kumeraho)

**DOI:** 10.1128/MRA.00629-21

**Published:** 2021-08-12

**Authors:** Monica C. Summers, Vincent V. Nowak, Michael J. Fairhurst, Jeremy G. Owen, Monica L. Gerth, Wayne M. Patrick

**Affiliations:** a Centre for Biodiscovery, School of Biological Sciences, Victoria University of Wellington, Wellington, New Zealand; University of Arizona

## Abstract

Kūmarahou (Pomaderris kumeraho) is a shrub endemic to New Zealand used in rongoā (traditional medicine). While studying the antimicrobial properties of kūmarahou, we isolated a new strain of Pseudomonas fluorescens, which we designated KF1 (for “kūmarahou flower 1”). Here, we report the complete genome sequence of P. fluorescens KF1.

## ANNOUNCEMENT

Kūmarahou (Pomaderris kumeraho) is a plant used in rongoā (traditional medicine) by the indigenous Māori of Aotearoa (New Zealand) (1, 2). While investigating the antimicrobial properties of this plant, we found a new strain that we subsequently identified as the Gram-negative bacterium Pseudomonas fluorescens. The bacterial strain was isolated from kūmarahou plant samples collected in the native New Zealand forest. Water extracts were made from flowers (10%, wt/vol) and spread onto potato dextrose agar. Several rounds of single-colony selection were undertaken until a clonal strain was isolated, as determined by uniform colony appearance and uniform growth rates upon restreaking. A stock was made by culturing the strain in potato dextrose broth at 22°C, adding glycerol (25%, vol/vol), and storing at −80°C.

In order to identify the strain prior to genome sequencing, a single colony was heated in 10 μl of sterile water at 95°C for 5 min and 1 μl of the lysate was used as the template for PCR. We used Q5 hot start high-fidelity master mix (New England BioLabs) and primers 515F and 806RB (3) to amplify the V4 region of the 16S rRNA gene. Initial denaturation occurred at 98°C for 30 s, followed by 25 cycles of 98°C for 5 s, 54°C for 10 s, and 72°C for 7 s and a final extension step of 72°C for 2 min. The resulting product was sequenced (Sanger sequencing; Macrogen Inc., South Korea), and a search with nucleotide BLAST (4) suggested that the strain was mostly likely to be in the P. fluorescens group.

Given the interest in P. fluorescens for its ability to promote plant health (5–7), we set out to sequence the genome of the new strain. The frozen stock was revived, and a single colony was used to inoculate 3 ml of potato dextrose broth. After 3 days of growth at 22°C with shaking, DNA was extracted using the Wizard genomic DNA purification kit (Promega, Gram-negative bacteria protocol). Two libraries were then prepared and sequenced by Macrogen Inc. The first library was prepared using the Illumina TruSeq DNA PCR-free kit, and paired-end reads 151 bp long were generated on an Illumina NovaSeq 6000 instrument. The second library was prepared using the PacBio SMRTbell template prep kit and sequenced on a PacBio RS II instrument using single-molecule real-time (SMRT) sequencing. This produced subreads of an average length of 9,642 bp with an *N*_50_ value of 14,172 bp. Other sequencing and assembly statistics are shown in Table 1.

**TABLE 1:** Assembly statistics and genome information for P. fluorescens KF1

Statistic or characteristic	Data for P. fluorescens KF1
Sequencing and assembly	
No. of Illumina reads	18,842,814
No. of PacBio reads	143,362
Total no. of Illumina bases	2,845,264,914
Total no. of PacBio bases	1,382,298,551
Avg coverage (×)	542
Genome description	
Genome size (bp)	6,957,239
GC content (%)	60.5
No. of genes (total)	6,306
No. of coding sequences (total)	6,220
No. of proteins	6,143
No. of pseudogenes	77
No. of rRNAs	16
No. of tRNAs	66
No. of ncRNAs^*a*^	4

a ncRNAs, noncoding RNAs.

The quality of the sequencing data was verified with FastQC v.0.11.7 (8). For the Illumina reads, the only warnings were GC content, some sequence duplication in forward, and some overrepresented GGG sequence in reverse. The PacBio polymerase read quality for subreads was largely above 0.85 and had an average FastQC Phred score of approximately 10. Several trimming parameters were trialed on the Illumina data using Trimmomatic v.0.36 (9), but raw reads were ultimately used in the final assembly, as this yielded the optimal result. *De novo* assembly was carried out using Unicycler v.0.4.7 (10) with the conservative setting and produced a single contig. The use of raw Illumina and PacBio reads was possible due to the high quality of the data and the hybrid assembly approach taken in Unicycler. Genome annotation occurred through the NCBI Prokaryotic Genome Annotation Pipeline v.4.13 (11) as part of the GenBank submission process. All programs were run under default parameters unless otherwise stated. Genome features are summarized in Table 1.

The genome sequence confirmed that we had isolated a novel strain of P. fluorescens, which we designated “kūmarahou flower 1” (KF1). The complete genome of P. fluorescens KF1 contains one circular chromosome of 6.96 Mbp, with a GC content of 60.5%. The phylogenetic placement of the strain was investigated using GTDB-Tk v.1.2.0 (12). This toolkit uses conserved marker genes to place a genome into the GTDB reference tree and, if it is assigned to a genus, compares the query genome to all species within that genus using fastANI (13). The highest average nucleotide identity was with P. fluorescens L321 (GenBank accession number CP015637), which was isolated from the miscanthus grass Miscanthus giganteus, based on its plant growth-promoting properties (6). While the average nucleotide identity match between the two strains is 99.27%, the L321 genome is smaller (6.6 Mbp), with a lower gene count (5,820 protein-coding genes).

### Data availability.

Raw reads for the project have been submitted to the NCBI SRA under the BioProject accession number PRJNA669298. The GenBank accession number for the complete genome is CP063233.
